# A Fusion Protein Consisting of the Vaccine Adjuvant Monophosphoryl Lipid A and the Allergen Ovalbumin Boosts Allergen-Specific Th1, Th2, and Th17 Responses* In Vitro*

**DOI:** 10.1155/2016/4156456

**Published:** 2016-06-01

**Authors:** Stefan Schülke, Lothar Vogel, Ann-Christine Junker, Kay-Martin Hanschmann, Adam Flaczyk, Stefan Vieths, Stephan Scheurer

**Affiliations:** ^1^Vice President's Research Group 1: Molecular Allergology, Paul-Ehrlich-Institut, Langen, Hessen, Germany; ^2^Division of Allergology, Paul-Ehrlich-Institut, 63225 Langen, Germany; ^3^Division of Microbiology, Paul-Ehrlich-Institut, 63225 Langen, Germany

## Abstract

*Background*. The detoxified TLR4-ligand Monophosphoryl Lipid A (MPLA) is the first approved TLR-agonist used as adjuvant in licensed vaccines but has not yet been explored as part of conjugated vaccines.* Objective*. To investigate the immune-modulating properties of a fusion protein consisting of MPLA and Ovalbumin (MPLA : Ova).* Results*. MPLA and Ova were chemically coupled by stable carbamate linkage. MPLA : Ova was highly pure without detectable product-related impurities by either noncoupled MPLA or Ova. Light scattering analysis revealed MPLA : Ova to be aggregated. Stimulation of mDC and mDC : DO11.10 CD4^+^ TC cocultures showed a stronger activation of both mDC and Ova-specific DO11.10 CD4^+^ TC by MPLA : Ova compared to the mixture of both components. MPLA : Ova induced both strong proinflammatory (IL-1*β*, IL-6, and TNF-*α*) and anti-inflammatory (IL-10) cytokine responses from mDCs while also boosting allergen-specific Th1, Th2, and Th17 cytokine secretion.* Conclusion*. Conjugation of MPLA and antigen enhanced the immune response compared to the mixture of both components. Due to the nonbiased boost of Ova-specific Th2 and Th17 responses while also inducing Th1 responses, this fusion protein may not be a suitable vaccine candidate for allergy treatment but may hold potential for the treatment of other diseases that require a strong stimulation of the host's immune system (e.g., cancer).

## 1. Introduction

Currently, conventional allergen immunotherapy (AIT) with allergen extracts is not convenient for patients due to a multiyear treatment regimen [1]. For some allergies, AIT is only partially efficacious and can be hampered by unwanted side effects. To improve AIT, novel vaccine candidates and accompanying adjuvants that increase efficacy while decreasing unwanted adverse-effects are needed [2].

In this context, the discovery of TLR-ligands with their intrinsic ability to induce robust innate immune responses was thought to hold great potential for the discovery and development of novel adjuvants. One of the best-characterized TLR-ligands is lipopolysaccharide (LPS), a cell wall component of Gram-negative bacteria that activates TLR4. Despite its strong immune-stimulatory potential, its use as an adjuvant is strongly limited due to its inherent toxicity [3]. Accordingly, nucleic acid-based TLR-ligands, such as CpG (TLR9), R848 (TLR7/8), or Poly I:C (TLR3), are good immune activators but are hampered in their clinical efficacy due to problems with both toxicity and stability* in vivo* [3].

To be able to take advantage of the strong immune-activating properties of TLR-ligands without the inherent toxicity, variants of TLR-ligands were generated by chemical modification which should retain most of their immune-stimulating properties [4]. One such adjuvant is the TLR4-ligand Monophosphoryl Lipid A (MPLA), a detoxified LPS-derivative. MPLA was derived from the LPS of* Salmonella minnesota* R595 by a series of organic extractions followed by mild acid and alkaline treatments [4]. This resulted in three distinct modifications compared to the parent molecule: (1) the removal of the core polysaccharide containing the O-antigen, (2) the removal of one phosphate group, and (3) one fatty acid chain [4].

Up to now, MPLA is the only TLR-ligand used as an adjuvant in licensed vaccines. Several vaccines including Fendrix® (hepatitis B), Cervarix® (human papillomavirus-16 and papillomavirus-18), RTS,S® (malaria) [5–7], and the allergen product Pollinex® Quattro (pollen allergies) [8] which contain MPLA as one component of more complex adjuvant systems have been licensed or are undergoing phase III clinical trials. Immunologically, MPLA has been repeatedly shown to induce a predominantly Th1-biased immune response [9]. The application of allergen therapeutics containing MPLA leads to isotype switching from allergen-specific IgE antibodies towards IgG_1_- and IgG_4_-dominated humoral immune response in humans [10].

While MPLA was reported to be less toxic and pyrogenic than LPS [11], the recent approval of several vaccine formulations adjuvanted with MPLA prompted us to initiate further detailed investigations of its adjuvant potential. In a previous study we showed that, in direct comparison to LPS, MPLA-stimulation induced similar but attenuated immune responses in several important immune cell types such as mouse epithelial cells, myeloid dendritic cells (mDCs), B and T cells, and human* ex vivo* isolated monocytes. Interestingly, MPLA was not able to activate either human or mouse mast cells [12].

After initially characterizing MPLA's immune-activating potential [12] we wanted to determine its potential as an integral part of an adjuvant : allergen fusion protein. In several experimental allergy models, such conjugates, for example, incorporating TLR5-, TLR7/8-, and TLR9-ligands, have been described to have beneficial immune-modulating properties by promoting Th1- and appropriate regulatory responses [13–16].

To this end, we chemically coupled MPLA to the model allergen Ovalbumin (Ova) and characterized the conjugate by SDS-PAGE and light scattering analysis. Subsequently, the immune-modulating properties of this MPLA : Ova fusion protein were investigated using mouse bone marrow-derived mDC and a coculture system of mDC and allergen-specific DO11.10 CD4^+^ T cells (mDC : DO11.10 CD4^+^ TC)* ex vivo* to directly compare what effect fusion of MPLA to Ova would have on the initiated immune response.

## 2. Methods

### 2.1. Coupling of MPLA and Ova

To activate MPLA (InvivoGen, Toulouse, France), it was dissolved in dried dioxane (Sigma) at a concentration of 50 mM and subsequently incubated at 37°C. 1,1′-Carbonyldiimidazole (CDI, Sigma, Steinheim, Germany) was added to a final concentration of 0.5 M and the mixture was incubated for 2 h at 37°C with stirring. Finally, dioxane was removed by the addition of diethyl ether and evaporation overnight. To couple the allergen to CDI-activated MPLA, EndoGrade Ovalbumin (Hyglos, Bernried, Germany) was dissolved in 10 mM sodium borate (pH 8.5) at 2.5 mg/mL and was subsequently used to dissolve the CDI-activated MPLA. After incubation for 48 h at 4°C with stirring, unconjugated MPLA was removed by extensive dialysis against PBS at 4°C for two days. The resulting MPLA : Ova fusion protein was characterized by SDS-PAGE and dynamic light scattering analysis.

### 2.2. SDS-PAGE

Chemically conjugated MPLA : Ova was compared to EndoGrade Ovalbumin (Hyglos) by SDS-PAGE according to the method described by Laemmli (cross linker C = 5%, total bis/acrylamide 15%) [17] under reducing conditions.

### 2.3. Dynamic Light Scattering Analysis

Dynamic light scattering analysis was performed using a Zetasizer Nano ZS (Malvern, Herrenberg, Germany). For light scattering analysis, 70 *μ*L of MPLA (1 mg/mL), MPLA : Ova (0.6 mg/mL), LPS (1 mg/mL), or Ova (1 mg/mL) in PBS was analyzed at room temperature. Three individual measurements per sample were performed and the mean frequencies (calculated as relative % in class) of hydrodynamic radii (*r*_H_) in nm were plotted.

### 2.4. *In Vitro* Generation of Mouse Bone Marrow-Derived Dendritic Cells

Mouse myeloid dendritic cells (mDCs) were generated as described previously [18]. Briefly, bone marrow cells (BMCs) were isolated from femur and tibia of BALB/c, mice and differentiated into mDCs using GM-CSF (R&D Systems, Minneapolis, USA). On day eight, mDCs were harvested for experiments.

### 2.5. Preparation and Stimulation of mDC and mDC : DO11.10 CD4^+^ T Cell Cocultures

Splenic CD4^+^ T cells were isolated from Ova-TCR transgenic DO11.10 mice using the CD4^+^ T Cell Isolation Kit (Miltenyi Biotec, Bergisch Gladbach, Germany). BALB/c mDCs (3.2 × 10^5^ cells/mL) were cultured alone or in combination with DO11.10 CD4^+^ T cells (8.0 × 10^5^ cells/mL, >95% purity) and stimulated with equimolar amounts of Ova, MPLA, MPLA mixed with Ova (MPLA + Ova), or MPLA : Ova (fusion protein) for 72 h. Subsequently, concentrations of IL-1*β*, IL-2, IL-5, IL-6, IL-9, IL-10, IL-12p70, IL-13, IL-17A, TNF-*α*, and IFN-*γ* in the supernatants were measured by BD OptEIA ELISA (BD Biosciences, Heidelberg, Germany) or Ready-SET-Go! ELISA Sets (eBiosciences, Frankfurt, Germany).

### 2.6. Statistical Analysis

The hypothesis of a significant higher cytokine secretion among all three concentrations used for stimulation was tested with a two-factorial analysis of variance (ANOVA) with factors stimulus (0.2, 1.0, and 5.0) and group (“MPLA + OVA” or “MPLA : OVA”). For statistical significant results the following convention was used: ^*∗*^ *p* value < 0.05, ^*∗∗*^ *p* value < 0.01, and ^*∗∗∗*^ *p* value < 0.001. The statistical analysis was performed with SAS/STAT software, version 9.4, SAS System for Windows.

## 3. Results

### 3.1. A Fusion Protein of MPLA and Ova Shows Noncovalent Aggregation

**Figure 1:**
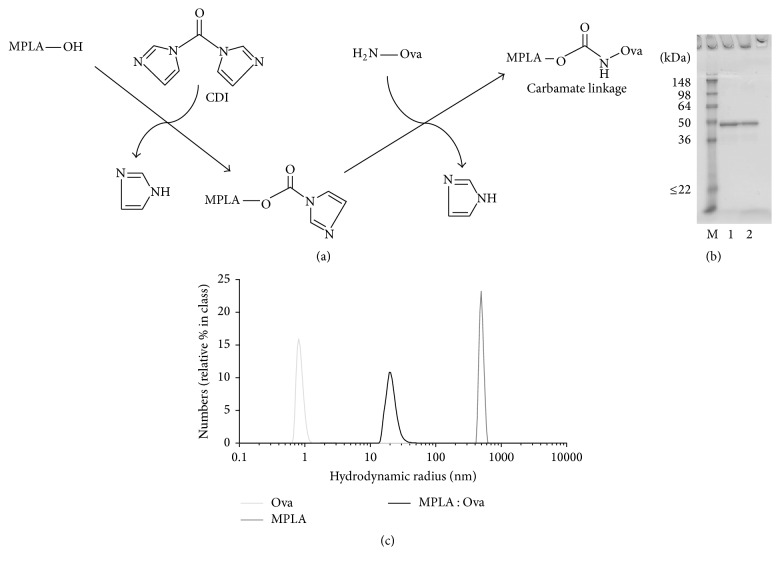
Generation of MPLA : Ova fusion protein. Applied chemical coupling strategy (a). Analysis of MPLA : Ova by reducing SDS-PAGE (b, M: molecular weight marker, 1: EndoGrade Ova, and 2: MPLA : Ova) and dynamic light scattering (c). For light scattering analysis, 70 *μ*L of MPLA (1 mg/mL), MPLA : Ova (0.6 mg/mL), LPS (1 mg/mL), or Ova (1 mg/mL) in PBS was analyzed at room temperature. Three individual measurements per sample were performed and the mean frequencies (calculated as relative % in class) of hydrodynamic radii (*r*_H_) in nm were plotted.

For the generation of the MPLA : Ova fusion protein, MPLA was conjugated to EndoGrade Ova using a carbonyldiimidazole linker in order to generate a stable carbamate linkage between both molecules (Figure 1(a)). Noncoupled MPLA was removed by extensive dialysis. The resulting MPLA : Ova fusion protein was characterized by SDS-PAGE and displayed a distinct band with an apparent molecular mass of 47 kDa (Figure 1(b)). Compared to Ova (apparent molecular mass of 45 kDa) this moderate shift of approximately 2 kDa suggests a coupling rate of one molecule of MPLA (molecular mass: 1.7 kDa) per molecule of Ova (Figure 1(b)).

Dynamic light scattering analysis determined the hydrodynamic radius of MPLA (*r*_H_ = 496 nm) to be larger than the radius of Ova (*r*_H_ = 0.9 nm, Figure 1(c)). This finding suggests aggregation in the MPLA preparation which is likely explained by the formation of micelle-like structures by the fatty acid chains of MPLA [19]. Here, the size of aggregates was reduced for the MPLA : Ova fusion protein (*r*_H_ = 20 nm), likely due to steric hindrance of micelle-formation induced by the fusion of Ova to MPLA (Figure 1(c)). However, compared to Ova alone, the hydrodynamic radius of the MPLA : Ova fusion protein was 22-fold enhanced in size, and no molecules with the hydrodynamic radius of either Ova or MPLA were detected in the MPLA : Ova preparation (Figure 1(c)). Taken together, these findings suggest both a complete coupling of the two molecules at a one-to-one ratio for the fusion protein and a complete removal of noncoupled MPLA by dialysis, resulting in a pure fusion protein preparation.

### 3.2. MPLA : Ova Boosts mDC-Derived Cytokine Secretion Compared to the Mixture of Both Components

**Figure 2:**
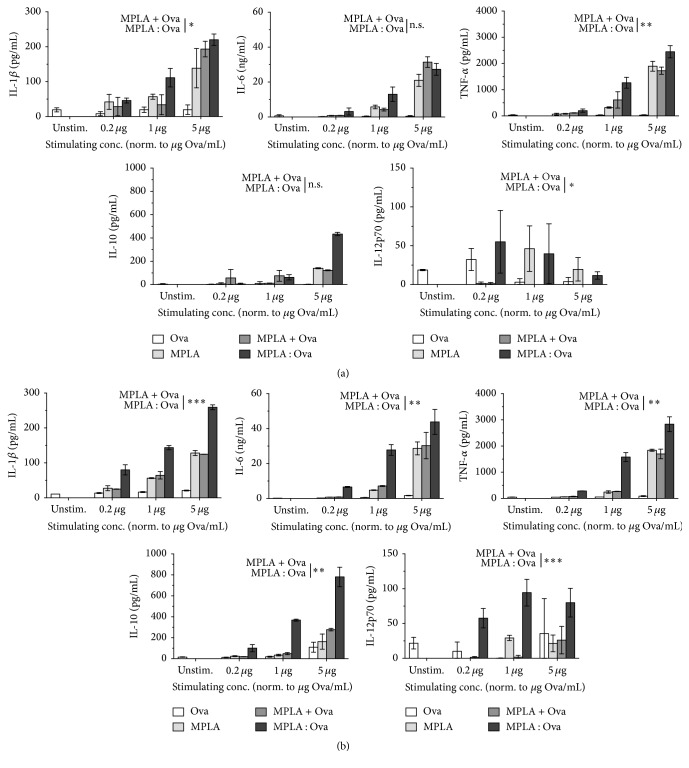
The MPLA : Ova fusion protein boosts mDC-derived cytokine secretion compared to the mixture of both components. Cytokine secretion determined from either BALB/c mDC (3.2 × 10^5^ cells/mL, a) or BALB/c mDC (3.2 × 10^5^ cells/mL) plus DO11.10 CD4^+^ T cell (8.0 × 10^5^ cells/mL, >95% purity) cocultures (b) stimulated with equimolar amounts of Ova (white bars), MPLA (light grey bars), MPLA + Ova (dark grey bars), and the MPLA : Ova fusion protein (black bars) for 72 h and analyzed by ELISA. ELISAs were performed using either BD OptEIA*™* ELISA (BD Biosciences) or Ready-SET-Go! ELISA Sets (eBiosciences). Data are mean results of two independent experiments ± SD. The hypothesis of a significant higher cytokine secretion among all three concentrations used for stimulation was tested with a two-factorial analysis of variance (ANOVA) with factors stimulus (0.2, 1.0, and 5.0) and group (“MPLA + OVA” or “MPLA : OVA”). For statistical significant results the following convention was used: ^*∗*^ *p* value < 0.05, ^*∗∗*^ *p* value < 0.01, and ^*∗∗∗*^ *p* value < 0.001. The statistical analysis was performed with SAS®/STAT software, version 9.4, SAS System for Windows.

**Figure 3:**
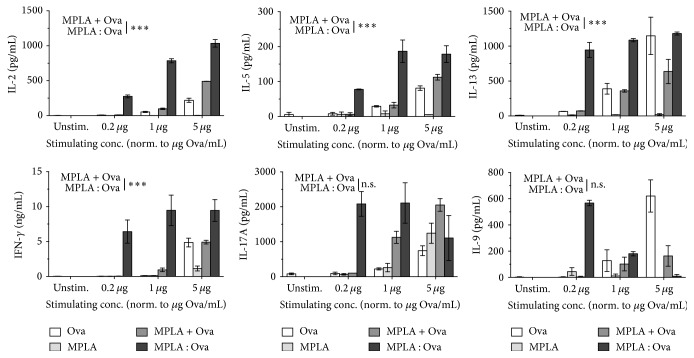
The MPLA : Ova fusion protein nonspecifically boosts Th1, Th2, and Th17 cytokine secretion from Ova-specific T cells. Cytokine secretion from BALB/c mDC (3.2 × 10^5^ cells/mL) and DO11.10 CD4^+^ T cell (8.0 × 10^5^ cells/mL, >95% purity) cocultures stimulated with Ova (white bars), MPLA (light grey bars), MPLA + Ova (dark grey bars), or MPLA : Ova (black bars) for either 24 h (IL-2) or 72 h (all other cytokines). ELISAs were performed using either BD OptEIA ELISA (BD Biosciences) or Ready-SET-Go! ELISA Sets (eBiosciences). Data are mean results of two independent experiments ± SD. Statistical analysis was performed according to Figure 2.

To investigate the potential immune-modulating properties of the fusion protein compared to both components alone or as a mixture we performed stimulation experiments using both myeloid dendritic cells (mDCs) alone (Figure 2(a)) and in coculture experiments with Ova-T cell receptor transgenic DO11.10 CD4^+^ T cells (Figures 2(b) and 3).

In mDC cultures, stimulated with the different constructs, application of the MPLA : Ova fusion protein resulted in increased secretion of IL-1*β*, IL-6, IL-10, and TNF-*α* (Figure 2(a), IL-1*β*: MPLA : Ova versus MPLA + Ova *p* = 0.0241, IL-6: *p* = 0.3447, IL-10: *p* = 0.2114, TNF-*α*: *p* = 0.0078). In contrast, stimulation with MPLA : Ova resulted in a dose-dependent decrease of IL-12p70 secretion, which was not observed for either component alone or the mixture of MPLA and Ova (Figure 2(a) IL-12p70: MPLA : Ova versus MPLA + Ova *p* = 0.0272).

Compared to the results obtained when stimulating mDCs alone, the levels of MPLA : Ova-induced cytokine secretion observed upon stimulation of mDC : DO11.10 CD4^+^ T cell cultures were either unchanged (TNF-*α* and IL-1*β*) or further increased (IL-6, IL-10, and IL-12p70, Figure 2(a) versus 2(b)). Therefore, when MPLA : Ova was added to mDC : DO11.10 CD4^+^ T cell cultures, the fusion protein induced a significantly higher cytokine secretion than equimolar amounts of either component alone or the mixture of MPLA and Ova (Figure 2(b)). Here, in direct comparison to the mixture of both components, the MPLA : Ova fusion protein significantly boosted both proinflammatory (IL-1*β*: MPLA : Ova versus MPLA + Ova *p* < 0.0001, IL-6: *p* = 0.0026, IL-12: *p* = 0.0001, TNF-*α*: *p* = 0.0015) and anti-inflammatory (IL-10: *p* = 0.0016) cytokine secretion.

Moreover, for the concentration corresponding to 1 *μ*g Ova per mL, MPLA : Ova induced 4-fold higher IL-1*β*, 4-fold higher IL-6, 6-fold higher TNF-*α*, 8-fold higher IL-10, and 53-fold higher IL-12p70 levels compared to the noncoupled mixture of MPLA + Ova (Figure 2(b)).

### 3.3. MPLA : Ova Boosts Th1, Th2, and Th17 Cytokine Secretion from Ova-Specific T Cells in a Nonbiased Way

In the next step we investigated the effect of MPLA : Ova-mediated mDC activation on the differentiation of Ova-specific CD4^+^ T cells (Figure 3). In addition to the significantly increased mDC-derived cytokine secretion (Figure 2), induced by the fusion protein compared to the controls, we observed the same effect for enhanced T cell-derived cytokine secretion in the applied coculture system (Figure 3).

In accordance with the results shown in Figure 2, at a stimulating concentration corresponding to 1 *μ*g Ova per mL, MPLA : Ova induced an 8-fold higher IL-2, 6-fold higher IL-5, 3-fold higher IL-13, 10-fold higher IFN-*γ*, 2-fold higher IL-17A, and 2-fold higher IL-9 secretion than the equimolar mixture of both components (Figure 3). Here, neither IL-2, IL-5, IL-13, IFN-*γ*, and IL-17A nor IL-9 secretion was detectable when mDCs were stimulated in the absence of Ova-specific CD4^+^ T cells (data not shown). Remarkably, at low concentrations (equivalent to 0.2 *μ*g/mL Ova) MPLA : Ova induced a 20-fold higher production of IL-17A in comparison to the equimolar mixture of MPLA and Ova, whereas at the highest applied concentration there was no difference between the different stimuli (Figure 3).

Of note, this effect was reversed for MPLA : Ova-induced IL-9 production, where stimulation with increasing amounts of MPLA : Ova resulted in a dose-dependent decrease in IL-9 secretion, while Ova alone dose-dependently induced IL-9 secretion (Figure 3, IL-9: MPLA : Ova versus MPLA + Ova *p* = 0.1691).

Finally, the MPLA : Ova fusion protein boosted Th1 (IFN-*γ*: MPLA : Ova versus MPLA + Ova *p* < 0.0001 and IL-2: *p* < 0.0001), Th2 (IL-5: *p* = 0.0004 and IL-13: *p* < 0.0001), and Th17 cytokine secretion (IL-17A, *p* = 0.1920Figure 3) from allergen-specific T cells without skewing the overall immune response in any particular direction.

## 4. Discussion

Herein we describe the generation and immunological characterization of a novel vaccine candidate consisting of the adjuvant MPLA and the model allergen Ovalbumin.

Adjuvant : allergen conjugates have several advantages over simple nonconjugated mixtures of both components: (1) they target the conjugate to the respective immune cells by binding to specific immune receptors (in this case TLR4 which may mediate both proinflammatory signaling and uptake). Upon binding to the target cell they (2) deliver the conjugated allergen to the immune cell in the context of the adjuvant-mediated immune cell activation which may influence allergen uptake, processing, and presentation [20]. Moreover, (3) adjuvant and allergen are simultaneously delivered to the same cell in a fixed molecular ratio, thereby preventing potentially detrimental bystander activation.

For this purpose, MPLA and Ovalbumin were coupled chemically via a stable carbamate linkage and the resulting fusion protein was characterized by SDS-PAGE and light scattering analysis. In SDS-PAGE, MPLA : Ova displayed a slight shift in molecular weight from approximately 45 kDa observed for Ova to approximately 47 kDa observed for MPLA : Ova. This moderate shift of approximately 2 kDa indicates a coupling rate of one molecule of MPLA (molecular mass: 1.7 kDa) per molecule of Ova.

Successful coupling of both molecules was further confirmed by light scattering analysis. With this assay we were able to demonstrate that the resulting MPLA : Ova fusion protein showed a single peak with a hydrodynamic radius of approximately 20 nm, which represents a 22-fold increase in size compared to nonconjugated Ova. Additionally, no molecules with the hydrodynamic radius of MPLA or Ova were detected within the MPLA : Ova preparation, demonstrating a complete removal of noncoupled MPLA by dialysis after coupling.

Previous studies investigating the effects of adjuvant : allergen fusion proteins, including TLR5-, TLR7-, and TLR9-ligands, on the modulation of allergen-specific immune responses demonstrated the potential for such conjugate vaccines to improve allergy treatment [13–16]. Studies by Kastenmüller et al. [15] and Filí et al. [16] describe allergen fusion proteins with TLR7- and TLR7/8-ligands as adjuvants.

Kastenmüller and colleagues reported a conjugate vaccine of a TLR7/8-ligand and Ova and showed this conjugate to elicit potent Th1-biased CD4^+^ and CD8^+^ T cell responses by activation and recruitment of dendritic cells to draining lymph nodes and the subsequent induction of type I interferon production [15]. In line with these results, Filí and coauthors described that the mite allergen nDer p 2 conjugated to a TLR7-ligand (4-(6-amino-9-benzyl-8-hydroxy-9H-purin-2-ylsulfanyl-)-butyric acid succinimidyl ester) stimulated IL-12 and IFN-*γ* production from monocytes and plasmacytoid DC and reduced allergic symptoms, while inducing allergen-specific IgG_2A_ antibodies in mice [16]. In this context, no induction of autoantibodies or Th17 cells was observed [16].

Moreover, Tighe and colleagues described the conjugation of a 22-mer CpG-motif, acting as a TLR9-ligand, to the major short ragweed allergen Amb a 1 [14]. In accordance with the results from the other adjuvant : allergen fusion proteins, this conjugate was shown to both induce Th1-biased immune responses in both naïve and sensitized mice and suppress IgE-induction after allergen-challenge [14].

In our own preliminary work we could show that prophylactic and therapeutic vaccination with a recombinant conjugate of the TLR5 agonist flagellin A (FlaA) from* Listeria monocytogenes* and Ova (rFlaA : Ova) was able to diminish Th2 responses in a mouse model of Ova-induced intestinal allergy [13]. Cocultures of mouse bone marrow-derived mDCs and CD4^+^ DO11.10 T cells demonstrated an IL-10-dependent reduction of Th2 and Th1 cytokine production upon stimulation with rFlaA : Ova but not with rOva and FlaA provided as a mixture [21].

When stimulating mDC with MPLA : Ova and the respective controls we observed an increased secretion of both proinflammatory (IL-1*β*, IL-6, and TNF-*α*) and anti-inflammatory (IL-10) cytokines. Here, in direct comparison of mDC stimulations with mDC : CD4^+^ TC coculture stimulations, overall levels of MPLA : Ova-induced cytokines were further increased in cocultures compared to the respective stimulation of mDCs alone. These results suggest that the mDC : TC interaction in the cocultures either further increased mDC-derived secretion (possibly by licensing effects of CD4^+^ T cells via mechanisms such as CD40-CD40L interaction) or induced additional production of the respective cytokines from Ova-specific T cells.

Unexpectedly in mDC : DO11.10 CD4^+^ T cell cocultures, when chemically fusing the TLR4 agonist MPLA to Ova, we observed a boost of Th2 cytokine (IL-5 and IL-13) production in such mDC : DO11.10 CD4^+^ T cell cocultures compared to equimolar amounts of MPLA + Ova. In addition, we observed an upregulation of both Th1 and Th17 cytokines IFN-*γ* and IL-17A as well as mDC-derived proinflammatory (IL-1*β*, IL-6, and TNF-*α*) and anti-inflammatory (IL-10) cytokines. Such strong APC activation and TC-derived cytokine boosts without distinct bias towards a defined T cell subtype (e.g., Th1-cells) are likely detrimental and can have significant consequences for both vaccine development and safety.

In contrast to this pattern, we observed a dose-dependent decrease of IL-9 secretion upon stimulation with the MPLA : Ova fusion protein while Ova alone or the mixture of MPLA + Ova dose-dependently induced IL-9 secretion. IL-9 stimulates cell growth and prevents apoptosis [21]; therefore, we believe that the observed reduction of IL-9 secretion upon stimulation with higher doses of MPLA : Ova represents a countermeasure to limit excessive cell activation and its potentially detrimental effects by this fusion protein. In line with our results the available literature describes the suppression of TC-derived IL-9 secretion by differentially activated DC: Rampal and colleagues reported that retinoic acid-monocyte-derived dendritic cells in the presence of TGF-*β*1 and IL-4 inhibited IL-9 and induced IFN-*γ* expression [22]. Concordantly, IFN-*γ* secretion was shown to inhibit Th9-differentiation [23]. Taking into account these results, the dose-dependent decrease of IL-9 secretion upon stimulation with the MPLA : Ova fusion protein may also be explained by the strong induction of other cytokines such as IFN-*γ* in higher stimulation concentrations. However, further investigations of this phenomenon and physiological relevance of MPLA : Ova-induced IL-9 secretion will need to be addressed in further* in vivo* studies.

In contrast to the strongly Th1-promoting TLR7 or TLR9 ligands, LPS was described to induce both Th1 and Th2 responses depending on either the applied dose [24] or the genetic background of the used organism [25]. The influence of genetic background on the capacity of LPS to induce either Th1 or Th2 responses was, for example, investigated by Soudi and colleagues [25]. They found that (in line with the well-described tendency of C57BL/6 and BALB/c to induce Th1 and Th2 responses, resp.) macrophages isolated from thioglycolate stimulated C57BL/6 mice produced more IL-17, IL-10, and IFN-*γ*, while BALB/c macrophages produced more TGF-*β* 1 and IL-4 when stimulated with LPS [25].

In our own previous work, when directly comparing LPS and MPLA for their capacity to skew Ova-induced T helper cell differentiation in BALB/c mDC : DO11.10 CD4^+^ TC coculture experiments we have demonstrated that MPLA was able to boost Ova-induced Th2-cytokine (IL-4, IL-5, and IL-13) secretion [12]. Interestingly, this effect was not observed upon coapplication of LPS and Ova [12]. Here, further studies are necessary to more clearly define the adjuvant capacity of MPLA in comparison to its parent molecule LPS. Also, while MPLA was shown to induce an immune deviation in favor of Th1 responses in grass pollen allergic donors [26] the differences in MPLAs adjuvant capacity in men versus mice are not yet fully clear and need further investigation. In line with this, the question whether the results obtained for the MPLA : Ova fusion protein in this study can be transferred to human DC : TC cocultures needs to be addressed in further studies.

In summary, we successfully generated a novel fusion protein consisting of the vaccine adjuvant MPLA and the model allergen Ova by chemical linkage. The generated fusion protein displayed a suggested coupling ratio of one molecule MPLA per molecule of Ova and was shown to aggregate, possibly mediated by the formation of micelle-like structures by the fatty chains of MPLA. Immunologically we observed that, compared to both components alone or as a mixture, the fusion protein boosted both mDC-cytokines as well as TC-derived Th1, Th2, and Th17 cytokine secretion without skewing the induced TC-differentiation in any particular direction.

Although the generated MPLA : Ova fusion protein may not be a suitable vaccine candidate for allergy treatment, due to the nonbiased boost of allergen-specific Th1, Th2, and Th17 responses, these findings open many new avenues for future research in the field of adjuvant biology, allergy, and immunology. Here, MPLA : antigen fusion proteins might hold potential for the treatment of other diseases which require a strong stimulation of the hosts immune system (e.g., cancer).

## 5. Conclusions

- A fusion protein of the TLR4-ligand MPLA and Ovalbumin (MPLA : Ova) was generated in a highly pure form with a coupling ratio of one molecule MPLA per molecule of Ova and without contaminations by either noncoupled MPLA or Ova.
- Immunologically, in mDC : DO11.10 CD4^+^ TC cocultures MPLA : Ova induced both stronger innate (mDC) and adaptive (Ova-specific TC) immune responses compared to the mixture of both components, boosting Th1, Th2, and Th17 TC-derived cytokine secretion.
